# Assessment as Learning in Medical Education: Feasibility and Perceived Impact of Student-Generated Formative Assessments

**DOI:** 10.2196/35820

**Published:** 2022-07-22

**Authors:** Ritu Lakhtakia, Farah Otaki, Laila Alsuwaidi, Nabil Zary

**Affiliations:** 1 College of Medicine Mohammed Bin Rashid University of Medicine and Health Sciences Dubai United Arab Emirates; 2 Strategy and Institutional Excellence Mohammed Bin Rashid University of Medicine and Health Sciences Dubai United Arab Emirates; 3 Institute for Excellence in Health Professions Education Mohammed Bin Rashid University of Medicine and Health Sciences Dubai United Arab Emirates

**Keywords:** self-regulated learning, assessment as learning, student-generated assessments, lifelong learning, medical education

## Abstract

**Background:**

Self-regulated learning (SRL) is gaining widespread recognition as a vital competency that is desirable to sustain lifelong learning, especially relevant to health professions education. Contemporary educational practices emphasize this aspect of undergraduate medical education through innovative designs of teaching and learning, such as the flipped classroom and team-based learning. Assessment practices are less commonly deployed to build capacity for SRL. Assessment *as* learning (AaL) can be a unique way of inculcating SRL by enabling active learning habits. It charges students to create formative assessments, reinforcing student-centered in-depth learning and critical thinking.

**Objective:**

This study aimed to explore, from the learners’ perspectives, the feasibility and perceived learning impact of student-generated formative assessments.

**Methods:**

This study relied on a convergent mixed methods approach. An educational intervention was deployed on a cohort of 54 students in the second year of a 6-year undergraduate medical program as part of a single-course curriculum. The AaL intervention engaged students in generating assessments using peer collaboration, tutor facilitation, and feedback. The outcomes of the intervention were measured through quantitative and qualitative data on student perceptions, which were collected through an anonymized web-based survey and in-person focus groups, respectively. Quantitative survey data were analyzed using SPSS (IBM), and qualitative inputs underwent thematic analysis.

**Results:**

The students’ overall score of agreement with the AaL educational intervention was 84%, which was strongly correlated with scores for ease and impact on a 5-point Likert-type scale. The themes that emerged from the qualitative analysis included prominent characteristics, immediate gains, and expected long-term benefits of engagement. The prominent characteristics included individuals’ engagement, effective interdependencies, novelty, and time requirements. The identified immediate gains highlighted increased motivation and acquisition of knowledge and skills. The expected long-term benefits included critical thinking, problem solving, and clinical reasoning.

**Conclusions:**

As a form of AaL, student-generated assessments were perceived as viable, constructive, and stimulating educational exercises by the student authors. In the short term, the activity provided students with a fun and challenging opportunity to dive deeply into the content, be creative in designing questions, and improve exam-taking skills. In the long term, students expected an enhancement of critical thinking and the inculcation of student-centered attributes of self-regulated lifelong learning and peer collaboration, which are vital to the practice of medicine.

## Introduction

### Background

Self-regulated learning (SRL) is a desirable student attribute that inculcates the habit of lifelong learning and is invaluable to budding health professionals [1]. SRL encourages adult learners to plan, implement, and evaluate their learning needs and outcomes. It works best as a supplement to traditional learning, with the adult learner increasingly taking charge of his own learning rather than passively receiving it. SRL has cognitive, metacognitive, behavioral, motivational, and emotional, or affective aspects that crosslink to make the end result either effective or not [2]. Most SRL models emphasize the development of this attribute in adult learners through preparatory, performance, and appraisal phases. In practice, SRL models can be stratified to become stage appropriate for the target student population, which, in turn, determines learning strategy and success [2].

Traditional theories and models of adult learning include instrumental learning theories, humanistic theories, transformative learning theories, social theories of learning, and motivational and reflective models [3]. In a review of their application to medical education, an Association for Medical Education in Europe guide proposes that student learners take charge of their learning through successive phases of dissonance, refinement, organization, and feedback, anchored by a learner-tutor nexus, wherein both roles are clearly defined for each phase [3]. Several teaching and learning activities can encourage the development of this quality. Flipped learning classrooms, simulation-based sessions with student-centered activities, and team-based learning are some teaching-learning formats used in preclinical medical education. In clerkship years, the learning context (eg, emergency room, inpatient bedside, or community practice) determines adult learners’ increasing reliance on SRL. These adapted formats promote higher-level cognition, as determined by the Bloom taxonomy (ie, application, analysis, evaluation, and synthesis of knowledge) expected of medical graduates, and are perceived by students as beneficial to learning [4].

Relatively little attention has been paid to developing active learning in undergraduate medical education by adopting learning techniques centered on assessment [5]. Feedback on formative and summative assessments aims to bridge identified learning gaps but remains a passive process, and its success depends on student follow-through. The best practice in assessment recommends that beyond the assessment *of* learning (AoL), which is summative and determines the achievement of outcomes, assessment *for* learning (AfL) through formative feedback and assessment *as* learning (AaL), which is learner-centric, are vital to enable cognitive and skill reinforcement [6]. In a critical review of the literature on learner-oriented assessment (LOA), Zeng et al [7] discussed the evolution of the Terrace-Kink traditional assessment pyramid from the traditional AoL at the bottom, followed by AfL and AaL at the tip of the pyramid. In this original version, AaL is at a higher level of achievement but assumes a minimal role. A rebalance of the original model shifts AaL to the base of the pyramid, thus making it foundational to and an enabler of learning, transferring AoL to the pinnacle as a definitive metric for achieving learning outcomes [7]. The authors proposed an adapted holistic framework for LOA by placing AaL, AfL, and AoL side by side with tutors and students partnering to achieve learning outcomes through innovative assessment practices. This composite framework could serve the overlapping purposes of learning, development, and certification.

The AaL framework places the contextual domain at the center of the AaL wheel for teaching and learning, supported by the societal, communication, and action domains [8]. It advocates the development of self-regulatory strategies by promoting cognition (ie, learning) and metacognition (ie, learning to learn). AaL works through student involvement in creating assessments, feed-forward on assessment results, and producing high-quality assessment tasks [9]. Student-generated assessments aim to encourage deep reading and demonstration of improved learning by creating questions that test higher-order thinking, thereby challenging students’ integration of disciplinary knowledge. Students can benefit from improved examination preparedness and performance by expanding the pool of formative questions [10]. Constructive curricular alignment, which involves the use of teaching designs that transparently demonstrate learning outcomes to both the faculty and the student aligned to appropriate assessment methods, can be enhanced through student-generated assessments [11]. This exercise can have other benefits, including collaborative work through peer engagement and receiving constructive criticism [12]. Although the intention to enhance student engagement and reinforce learning abilities and styles through assessment is desirable, it is also essential to hear the student’s voice by exploring their perceptions of such an educational intervention. Active student engagement and learner agency can only be ensured when they perceive the benefits of an educational intervention, both in immediate learning and in enhancing SRL [13].

### Objectives

As such, this study aimed to explore, from the learners’ perspective, the feasibility and perceived learning impact of student-generated formative assessments. Accordingly, the research questions of this study are as follows:

1. To what extent did the students agree that the experience of contributing to formative assessment was manageable (in terms of difficulty level) and impactful, and in what ways were the perceived ease and impact associated?
2. How do the students describe the experience of contributing to formative assessment?
3. What are the lessons learned from the firsthand experience of having students contribute to formative assessments?

## Methods

### Ethics Approval

The ethics approval for this study was granted by the Mohammed Bin Rashid University institutional review board (MBRU-IRB-2019-026). Informed consent was obtained from all the participants. All methods were performed in accordance with relevant guidelines and regulations. Consent for publication was not applicable as there are no individual details, images, or videos.

### Research Design

This study relied on a convergent mixed methods research design [14], which is commonly used in health professions education research [15-17]. The strength of this multiphase research design lies in its potential to capture a holistic perspective of the subject matter. Instead of focusing on the generalizability of the generated results, the emphasis was on their transferability to other similar contexts. This research design is expected to generate sufficient in-depth insights [18,19]. For this purpose, a survey designed by the research team in consensus (for this study) was assembled to capture quantitative and qualitative data on undergraduate medical students’ perceptions of their engagement in developing formative assessments. This unique educational intervention of student-centered assessment (ie, AaL) was implemented in a required 3-credit course entitled Pathologic Basis of Diseases. Quantitative and qualitative data were analyzed independently and then merged using a joint display analysis. As such, the integration of data is meant to raise the study’s robustness and validity of the generated findings [20].

### Educational Context of the Study and Participants

This study was undertaken at the Mohammed Bin Rashid University of Medicine and Health Sciences, Dubai, United Arab Emirates, on a single cohort of students of a 6-year medical undergraduate program (MBBS) following a spiral curriculum and divided into 3 sequential phases: foundational basic sciences, preclinical sciences, and clerkship. Phase 1 takes place over the first academic year and introduces students to basic concepts in medicine, whereas phase 2 covers academic years 2 and 3, where teaching is organized around body organ systems and integrated with clinical medicine. Years 4 to 6 constitute phase 3. During the first 2 years of this phase, students undergo clinical placements or rotations, with the final academic year taking the form of an internship. The study cohort comprised 54 second-year students (academic year 2019-2020) beginning phase 2 of the undergraduate medical curriculum.

### Description of the Intervention

**Figure 1:**
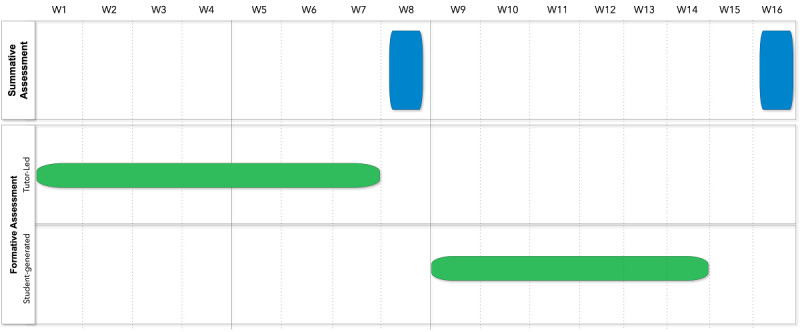
Study overview. Tutor-driven formative assessments in the first half of the semester were followed by a midsemester in-course summative assessment. The student-generated formative assessments in the second half of the semester were followed by the final summative assessment of the course. W: week.

The course under investigation was the medical students’ introduction to pathology. During the first 6 weeks of the semester, the students were provided with weekly formative assessments generated by the pathology faculty teaching the course, followed by feedback sessions to reflect upon identified points of strengths and weaknesses. An in-course summative assessment (weighing 40%) was administered midsemester in week 8. The students generated formative assessments in a multiple-choice question (MCQ) format between weeks 9 and 14. The end-semester summative assessment (weighing 60%) was conducted in week 16 (Figure 1).

**Figure 2:**
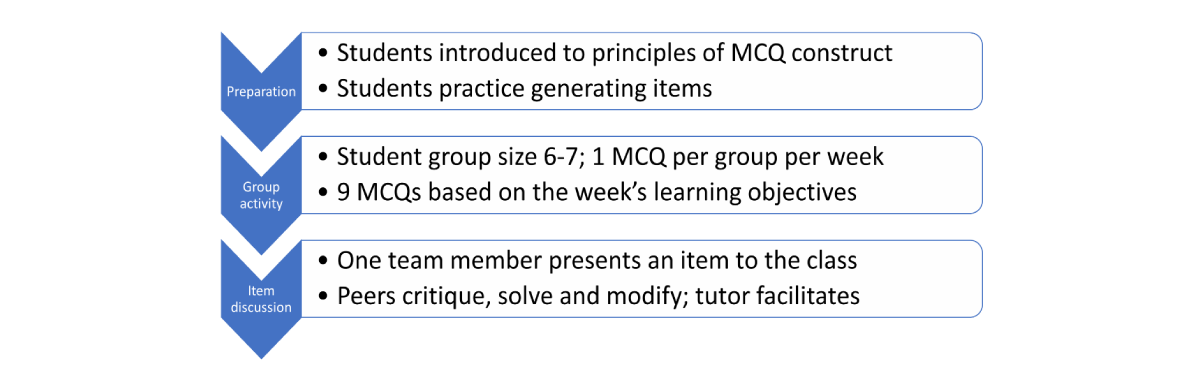
The educational intervention: assessment as learning. The educational intervention comprised weekly student-generated multiple-choice questions (MCQs) created through peer collaboration and supplemented by peer critique and review, tutor moderation, and feedback.

Students were first guided in the principles of MCQ construction by a professor of pathology, who coordinated and taught the course and was also a chair of assessment in the college. A total of 9 groups comprising 6 students each created 1 MCQ per week on the ongoing week’s learning outcomes and lesson objectives. The resultant 9 MCQs were discussed the following week at the allotted time, supplemented by tutor-generated questions. One representative per group presented their MCQ and invited critical and constructive comments from peers. The professor tutor moderated the discussion and provided feedback on the constructs and content (Figure 2).

### Data Collection

Data were collected using a survey designed specifically for this study (Textbox 1). The survey comprised 2 segments. The first segment was a 5-point Likert-type scale (1=strongly disagree, 2=disagree, 3=neutral, 4=agree, and 5=strongly agree) across 10 components, all of which were mandatory to respond to. Components 1 to 5 were meant to evaluate the ease of contributing to the development of formative assessments. Components 6 to 10 were designed to capture students’ perception of the impact of contributing to the development of formative assessments. It was mandatory to respond to all 10 components. The reason for which these 2 variables (ie, *ease* and *impact*) were pinpointed is that it is established (in alignment with the theories of behavioral change) that the students’ perceptions of this educational intervention’s barriers to its implementation (ie, ease) and benefits (ie, impact) significantly affect its effectiveness (in terms of maximizing learning) [21,22]. This link has been further reinforced in research on SRL [13].

**The components of the quantitative segment of the tool adapted for this study.**

**Ease of contributing to the development of formative assessments**

1. The exercise was fairly simple (exercise fairly simple).
2. The exercise enabled me to become more competent at developing questions (competence in developing questions).
3. Effectively undergoing the exercise required that I get out of my comfort zone (out of my comfort zone).
4. I am willing to repeat this exercise for other courses (willingness to repeat the exercise in other courses).
5. Contributing to the creation of formative assessments adds value to the learning experience (adds value to learning).

**Impact of contributing to the development of formative assessments**

1. The exercise raised my capacity to understand the respective course material (capacity to understand course material).
2. Developing questions improved my knowledge of the subject matter (knowledge of subject matter).
3. The exercise developed my critical thinking (critical thinking).
4. The exercise raised my capacity to effectively answer relevant questions (capacity to answer relevant questions).
5. In and of themselves, the exercise and the generated in-class feedback and reflections on the created questions improved my capacity to associate the respective basic science concepts with their medical application (ie, clinical correlation skills).

The participants were given the option of qualitatively elaborating their responses to each of the 10 components. The second section of the survey entailed an open-ended question that was meant to solicit any additional reflective qualitative data using the following open-ended question: “Do you have any further remarks on your engagement with developing formative assessments? If so, please indicate them below:”

The survey was initially developed by the pathology faculty teaching the course and underwent face and content validity checks. The face validity test was conducted by a team of professionals, comprising the coordinator of the respective course, the chairperson of the College of Medicine Student Assessment and Progression committee, an expert in medical education, and a staff member of the unit that handles the respective university’s Quality Assurance and Institutional Effectiveness portfolio. They reviewed the tool to assess the clarity, comprehensibility, and readability of the questions and the flow through which they were presented. Subsequently, content validity was assessed by randomly selecting 5 students from the preceding cohort of the same program. They were invited to a classroom where they were asked to write down their interpretation of each of the components within the first segment, as well as the questions in the second segment of the survey. These responses were reviewed by the abovementioned team. A consensus was reached that other than minor language changes, the survey was ready to be administered.

Participation in this data collection initiative was voluntary. The students’ privacy and data confidentiality were protected, and no personal identifiers were recorded. The survey was assembled on the web throughout May 2020 using Microsoft Forms. Each study participant was serially assigned a unique identification number (1-27).

### Data Analysis

#### Quantitative Analyses

Quantitative data were analyzed using SPSS (version 25.0; IBM Corp) for Windows. For each of the 10 quantitative components (measured with a 5-point Likert-type scale), the mean and SD were calculated. Subsequently, the percentage of the mean for each component was calculated by dividing the respective mean by 5 (as it is the maximum possible value) and multiplying it by 100, which determines where the 10 corresponding values lie on the predefined scale. An overall score of the agreement for all components (ie, the total of the means of all 10 components) was computed, along with an independent score for each of the 2 segments of the tool: ease and impact (ie, the total of the means for each of the 2 groups of 5 components). The mean and SD were calculated for all 3 scores.

As the scale used to capture the perception of the participants was tailor-made for this study, the validity tests of Cronbach α and the principal component analysis of the Kaiser-Meyer-Olkin and Bartlett test were performed to check the internal consistency and external variance, respectively, of the designed tool.

To select appropriate means of correlating the variables, a test of normality was conducted for each of the 10 components and for the following 3 scores: overall, ease, and impact. The data for each of the 10 components and the ease and impact scores were not normally distributed. The overall agreement score was normally distributed (*P*=.38). Accordingly, a matrix of bivariate correlations was developed using the Spearman test to assess the extent to which the 3 scores related to each other and their components.

#### Qualitative Analysis

Qualitative data analysis began after the conclusion of the data collection phase. The data were analyzed (based on constructivist epistemology) by 2 researchers (RL and FO) using thematic analysis following a 6-step framework [23,24]. As such, the researchers began by familiarizing themselves with the data. Each of them reviewed the data set independently while writing down notes about key observations. They then convened to discuss their notes. The next step revolved around generating initial codes for prominent patterns identified after the initial step of examining the data set. The third step, which was the most extensive, involved searching for the themes. This required the development of several iterations of mind maps, where the manner in which the generated codes related to one another was visually presented. The fourth step included a review of themes to ensure that there was sufficient similarity between all text fragments placed within the same group while ensuring that there were enough dissimilarities across the groups to differentiate them from one another. The fifth step was defining and naming the generated themes. The last step involved reporting on the results of the qualitative analysis, which was done based on recently published standards of reporting on qualitative analysis integral to mixed methods research design [25].

#### Joint Display Analysis

The quantitative and qualitative data were then mapped onto each other through the iterative process of joint display analysis [18]. Integration was meant to reveal where the findings confirmed or built upon each other. was also able to shed light on where the findings contradict each other. Therefore, meta-inferences were generated [14].

## Results

### Quantitative Analyses

Of the 54 students, 27 responded (ie, response rate of 50%). The reliability score of Cronbach α for the tailor-made evaluation tool that captured the students’ perceptions (ie, 10 components) was .84. The percentage of the total average of the overall score of agreement was 84%, somewhere between *agree* and *strongly agree*, as per Table 1.

The sampling was determined as adequate with a Kaiser-Meyer-Olkin close to 1. In addition, according to the Bartlett test of sphericity, the null hypothesis was rejected with an identity matrix in which all diagonal elements were 1 and all off-diagonal elements were 0. As such, the principal component analysis (along with the corresponding eigenvalues) showed that 75.2% of the variance across the 10 components could be explained by the instrument as a whole. This means that the instrument was reliable and valid for measuring what it intends to measure.

**Table 1:** Output of descriptive quantitative analysis.

Component	Values, mean (SD)	Percentage of the mean (%)	Category
1	4.19 (0.736)	83.8	Agree to strongly agree
2	4.22 (0.641)	84.4	Agree to strongly agree
3	3.41 (1.083)	68.2	Neutral to agree
4	4.04 (1.055)	80.8	Agree
5	4.52 (0.643)	90.4	Agree to strongly agree
6	4.56 (0.577)	91.2	Agree to strongly agree
7	4.33 (0.679)	86.6	Agree to strongly agree
8	4.19 (0.736)	83.8	Agree to strongly agree
9	4.30 (0.724)	86	Agree to strongly agree
10	4.26 (0.813)	85.2	Agree to strongly agree
Score of ease	15.85 (2.231)	79.3	Agree
Score of impact	26.15 (3.45)	87.2	Agree to strongly agree
Overall score of agreement	42 (4.907)	84	Agree to strongly agree

### Correlational or Inferential

As illustrated in Table 2, the overall score of agreement was significantly influenced by the perception of the students regarding all components except for component 3, “Effectively undergoing the exercise required that I get out of my comfort zone” (*P*<.001). Moreover, all 3 scores: overall, ease, and impact, correlated with each other (*P*<.001 for overall and ease, and overall and impact & *P*=.01 for ease and impact).

**Table 2:** Matrix of bivariate correlations.

Component	*P* value												Correlation
	1	2	3	4	5	6	7	8	9	10	Score		
1													
	.99	.44^a^	−.23	.62^a^	.32	.13	.42^a^	.38^a^	.44^a^	.34	.59^a^	Coefficient	
	—^b^	.02^a^	.24	.001^a^	.11	.53	.03^a^	.049^a^	.02^a^	.08	<.001^a^	Significant	
2													
	—	.99	.03	.33	.29	.21	.48^a^	.48^a^	.53^a^	.39^a^	.66^a^	Coefficient	
	—	—	.87	.09	.14	.30	.01^a^	.01^a^	.004^a^	.046^a^	<.001^a^	Significant	
3													
	—	—	.99	.11	−.13	.05	−.08	.12	.007	.02	.22	Coefficient	
	—	—	—	.60	.52	.81	.69	.56	.97	.93	.28	Significant	
4													
	—	—	—	.99	.27	.12	.48^a^	.40^a^	.30	.36^a^	.63^a^	Coefficient	
	—	—	—	—	.18	.54	.01^a^	.04^a^	.13	.06^a^	<.001^a^	Significant	
5													
	—	—	—	—	.99	.57^a^	.58^a^	.65^a^	.41^a^	.71^a^	.64^a^	Coefficient	
	—	—	—	—	—	.002^a^	.002^a^	<.001^a^	.03^a^	<.001^a^	<.001^a^	Significant	
6													
	—	—	—	—	—	.99	.71^a^	.63^a^	.49^a^	.60^a^	.60^a^	Coefficient	
	—	—	—	—	—	—	<.001^a^	<.001^a^	.01^a^	.001^a^	<.001^a^	Significant	
7													
	—	—	—	—	—	—	.99	.74^a^	.60^a^	.63^a^	.81^a^	Coefficient	
	—	—	—	—	—	—	—	<.001^a^	.001^a^	<.001^a^	<.001^a^	Significant	
8													
	—	—	—	—	—	—	—	.99	.63^a^	.76^a^	.85^a^	Coefficient	
	—	—	—	—	—	—	—	—	<.001^a^	<.001^a^	<.001^a^	Significant	
9													
	—	—	—	—	—	—	—	—	.99	.67^a^	.75^a^	Coefficient	
	—	—	—	—	—	—	—	—	—	<.001^a^	<.001^a^	Significant	
10													
	—	—	—	—	—	—	—	—	—	.99	.78^a^	Coefficient	
	—	—	—	—	—	—	—	—	—	—	<.001^a^	Significant	

^a^Correlations that revealed significance, as defined by the *P* value.

^b^Not applicable.

### Qualitative Analyses

**Figure 3:**
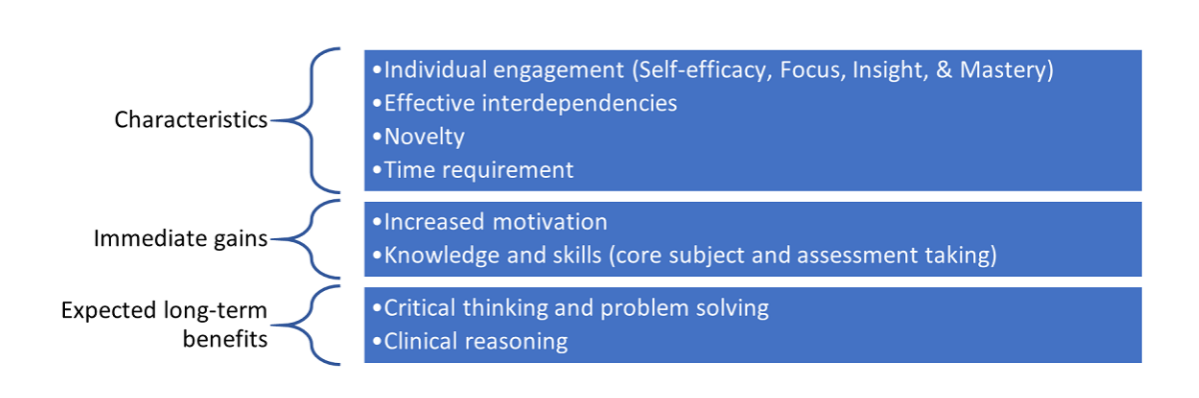
The conceptual framework of the study. Prominent characteristics emerged from students’ perceptions of self-generated formative assessments demonstrating immediate gains and expected long-term benefits and validating the educational intervention’s utility toward assessment as learning.

The analysis of the qualitative data capturing the students’ perceptions resulted in 3 interrelated themes: prominent characteristics, immediate gains, and expected long-term benefits of their engagement in preparing the formative assessment (Figure 3).

#### Theme 1: Prominent Characteristics

This theme included text fragments that referred to how the students characterized the program and what stood out to the students as the variables upon which the activity’s success relies. This included variables such as immersing oneself in the experience:

...it was not very easy since one needed to concentrate and focus a lot to develop MCQs...

Participant 23

It was clear that the participants needed to form effective interdependencies with colleagues:

...we needed to come up with questions related to our own learning...it was a team effort...discussing the questions, among each other, enabled us to develop a better idea as to what would constitute good distractors...the variety of perspectives was useful, of course...

Participant 11

Some students highlighted that teamwork inherent to the exercise and ensuring that all team members were equally engaged was challenging:

...the same people, within our team, kept on generating the questions. Not all the team members contributed equally; some members did not provide any input...we were able to eventually address this challenge...I needed to converse more with some of my colleagues whom I do not usually have the opportunity to speak to...

Participant 14

...some of the group members did not bother to do their job in developing questions, which caused some frustration within the team...

Participant 22

Students believed that engaging in the experience enabled them to develop the necessary insights and mastery or proficiency in preparing formative assessments. This belief, coupled with focusing on the exercise at hand, helped them develop their self-efficacy:

...we are expected to generate the MCQs soon after we learn a new concept. This required that we look-up key terms and additional information related to the respective concept. As part of preparing for the MCQs, we needed to come-up with distractors. We needed to really understand the content to be able to do the task...

Participant 3

...to develop the capacity to create our own MCQs and share them with other students...

Participant 11

This theme also included text fragments that showed that the students were aware that the experience was novel and that they had to go through a learning curve:

...it was surely a new experience for me; we were given the opportunity to view the exam from the examiner point-of-view, from the perspective of the person forming the MCQs. It felt really good...

Participant 3

...it was fairly simple, but developing more elaborate questions was more challenging...

Participant 4

...it is the details that matter and that was a bit difficult, at first. Trying to discern two similar topics, while thinking of sequential order and associated elements, and formulating possible choices, among which the “best” answer, were the steps that required extra effort...

Participant 8

...formative assessments allow us to test our understanding of concepts without the burden of having to perform well in terms of a test or a grade which gives us more opportunities to make mistakes and to learn from them...

Participant 11

...I am not familiar with such exercise so I was getting out of my comfort zone, but I would say, in a positive way...

Participant 19

The students also highlighted how the exercise required time investment:

...however, since we were given enough time to do it, it was good...

Participant 23

#### Theme 2: Immediate Gains

This theme encapsulated all text fragments that referred to what the students gained upon completing the experience. In general, most students expressed excitement and were happy to have gone through this experience:

...finding sensible, reliable distractors became a “hobby” when forming MCQs...It was a valuable experience, and a good exercise. Plus, it was fun...

Participant 3

...it was an interesting and helpful exercise...

Participant 15

It was evident to the students that they had gained ample knowledge and skills from their experience:

...it was both beneficial for our learning and interesting for us since we got to see how much work it actually takes to formulate proper questions...It was a very useful and interesting task...

Participant 27

The students referred to learning that occurred in relation to the core subject (ie, pathology):

...this exercise enabled us to effectively learn the core concepts of pathology...

Participant 3

...it allowed for additional practice on the learning material...

Participant 8

...this exercise covered some parts that I might have missed or did not fully comprehend, at first...

Participant 14

...in order to structure a question, I had to gain good understanding of the topics, so it was really helpful...

Participant 20

Enhancing the knowledge and skills around assessment taking was also apparent to the students:

...we were required to prepare a test-like question from the preceding weeks material...we learned about the types of questions and of possible answers that are commonly used which enabled me to approach the course material in a different role...

Participant 8

...it really enhanced how I tackle questions and how I think when answering questions...

Participant 14

...we got to understand how the examiner thinks; this is a good skill that is useful for us to have when revising the required content prior taking any one exam...

Participant 15

#### Theme 3: Long-term Benefits

This theme included text fragments that referred to the gains that the students expected to materialize over time from this experience (eg, critical thinking and clinical reasoning):

...this process gave me the opportunity to change my learning style...to create a question, one needs to approach the topic differently; this reinforces one’s understanding of the topic and equips the students with transferable skills...

Participant 8

...pathology clinical are really essential and shade huge light on the grey area that connect the aetiology/pathology to clinical manifestation...

Participant 14

...I learned how to figure-out what to focus on, what the important parts of any lecture is...I think it was great; it gave us insight as to what the actual assessment will be like and helped prepare us for the In-Course Assessment...

Participant 24

...my question writing skills, which require ample of critical thinking and problem-solving skills, improved since I had to formulate questions that were advanced...this all was so beneficial to my learning...

Participant 27

### Data Integration

The convergence of the quantitative and qualitative data resulting in the meta-inferences is shown in Table 3. Quantitative inputs derived from Textbox 1 and Table 1 were mapped to themes 1 to 3 of the qualitative data as the perceptions of ease and impact on the individual and the group. The meta-inferences were characterized as strengths, weaknesses, challenges, and opportunities, which provide an opportunity to consolidate and build on gains and remedy weaknesses in innovation. The identified strengths of the educational intervention were well matched in the quantitative and qualitative perceptions of the students, except for the interesting qualitative description that learning through assessment was more enjoyable as the exercise had a gamification aspect. However, weaknesses were entirely identified in qualitative responses and not through quantitative scores, such as time management and disruptive peer dynamics. Among the perceived challenges and opportunities, qualitative inputs provided additional insights that enhanced available quantitative data, such as the novelty of the experience. Overall, the qualitative data lend themselves to actionable evidence, which considerably enhances the conclusions of the study.

**Table 3:** Joint display: output of integrating quantitative data with qualitative data.

Quantitative (Textbox 1 and Table 2^a^)	Meta-inferences	Qualitative (themes 1-3)	
		Individual	Group
Questions 1-2, 6, and 9-10: agree to strongly agree	Strengths	Simple and easy to make Revision of content Examination-taking skills No stress to score	Peer reinforcement Stratification and sequencing of learning Gamification
The qualitative method provides insights not revealed by quantitative survey^b^	Weaknesses	Time consuming (but manageable in the time provided)^b^	Repeat questions in groups Unequal participation by peers (teamwork)^b^
Question 3: neutral to agree; question 4: agree to strongly agree; question 10: agree to strongly agree	Challenges	Out of comfort zone Required focus	Willingness to repeat the exercise To create questions of higher-order thinking
The qualitative method provides insights not revealed by quantitative survey^b^ (questions 6-10): agree to strongly agree	Opportunities	A novel method of learning^b^	Critical thinking Problem solving

^a^Quantitative analyses (Table 1).

^b^Additional insights from qualitative data.

## Discussion

### Principal Findings and Comparison With Prior Work

This educational intervention to promote SRL provides insights into engaging students in AaL exercises. The design of the intervention mirrored the phases of preparation, implementation, and appraisal, which were well-illustrated in an insightful meta-analysis by Panadero [2] of 6 individual SRL models proposed by Boekaerts, Efklides, Haldwin, Pintrich, Winne and Hadwin, and Zimmerman, respectively. The study focuses on *appraisal* of this form of SRL from a student’s viewpoint capitalizing on the strengths of the mixed methods approach. In the process, the quantitative measurement of the ease and impact of AaL was considerably enhanced and supplemented by students’ qualitative inputs. The latter provided prominent characteristics of the experience, as well as short- and long-term impacts. Integration of the mixed methods data on the ease and impact of the AaL intervention provided robust metrics (quantitative) on the strengths, weaknesses, challenges, and opportunities amplified by incisive observations (qualitative). Certain experiences could only be captured by subjective expressions in the students’ own words. The positive inputs included the novelty of the experience and the gamification effect, which enhanced the enjoyment of learning. There was also a useful critique of unequal levels of peer contribution, quality of questions, or repetition in some groups. In the following paragraphs, these observations are discussed in relation to shared experiences from the published literature.

This study achieved a partnership between students and tutors, as emphasized in the holistic approach to LOA [7]. This study sheds light on how, from a constructivist perspective, assessment can be leveraged to drive students’ learning. Constructivism implies the learners’ central role in taking charge of their learning, gaining insights into learning gaps, and developing ways of improving learning. AfL can be a significant component of this self-regulatory mechanism but often relies on feedback after formative assessments that remain tutor driven, focused, and directive. However, in AaL, students assume control by dominating the learning process’s discourse and producing a self-regulatory and self-productive identity [6]. Students set goals, monitor progress, and reflect on learning prospectively, not retrospectively, as in formative assessments.

In this study, the design of the AaL innovation addressed the student-centered communication and action domains of the AaL wheel [8]. Evaluating students’ perceptions of AaL implemented in the course of an MBBS program proved to be rewarding. In this study, students’ qualitative reflections on undertaking assessment creation were characterized as short- and long-term gains. Students’ scores on overall agreement of engagement with designing assessment and the related ease and impact were all considerably high, with significant correlations among all 3 scores. Similarly, in a previously assembled survey inquiring about a medical student–generated question bank at the University of Manitoba, Winnipeg, Manitoba, Canada, 91% of students reported satisfaction with their engagement in developing questions [12].

The quantitative results of this study showed that the only component that was not statistically associated with the students’ overall agreement with the experience was that the exercise required them to leave their comfort zones. Qualitative inputs showed that the experienced unease was favorably perceived as an enabling challenge along the same line. The idea that leaving one’s comfort zone can be of added value is well-established in the literature [26]. In a US dental undergraduate program, a study on student-generated MCQ items reported that the students were able to prepare a higher cognitive level of questions than the instructor [27]. The students perceived the intervention as contributing to their learning. Thus, student creation of assessments provides a unique opportunity for learners within a developmental framework of assessment [28].

In this study, the students specifically expressed their realization of the added value of assessment-enhanced learning toward the core content of the specific course. According to the students, this happens when tasked with preparing questions by increasing their focus on the subject matter and by the requirement of viewing it from a different perspective. They were surprised by how their efforts to create questions contributed to exam preparedness and insight into the examiner’s viewpoint. One could extrapolate that this would reduce the stress of exam preparation at the end of the semester. The development of higher-order thinking is best achieved through inquiry and investigation, applying knowledge to new situations and problems, producing ideas and solutions, and collaborative problem solving [29].

The high level of agreement reported in this study was related to students’ perceptions of the value of learning. This, in turn, encouraged students to invest time and effort, positively reinforcing the link to the perceived learning impact of the exercise. Students commented that creating questions weekly promoted regularity in their reading, reflecting, and revising habits. The literature on the subject matter indicates contradictory findings. In a study on undergraduate students who generated MCQs in the fourth year of the pathology course of a New Zealand medical school, students could create cognitively challenging MCQs. However, they did not find the task of educational value [30]. The students engaged well with the peer-wise platform for question creation but did not offer good peer feedback. In contrast, in another study involving second-year biomedical sciences students (n=107), perceptions of student-authored assessments in a biochemistry course demonstrated an eagerness and the generation of a large repository of relevant and good-quality MCQs [31].

An example of student-generated formative assessments specifically targeting competency-based progression was illustrated in a multicenter pilot study in German medical schools [32]. A core team of 17 students from the third to ninth semesters drawn from 17 universities was trained on MCQ generation and review, contributing 118 MCQs to a 144-item assessment based on a preagreed competency blueprint. It was administered to 469 students from 8 medical schools. The items were of high quality with higher-order thinking and generated high test reliability. However, student authors seemed to favor item generation on theoretical and practical skill competencies over scientific and communication skills competencies. The examinees perceived it more as an opportunity for feedback rather than a learning experience.

Another unexpected but beneficial aspect highlighted by students is the perceived “gamification effect” of the exercise. During moments of relaxation, tossing around distractors became a second habit to them as an intellectually entertaining tool. According to Gray [33], creativity is the basis of critical thinking and always involves a degree of playfulness: “the critical thinker plays with ideas...to see what happens and to explore consequences.” The development of such instinctive and enjoyable learning through play can have a long-lasting impact, sustaining self-learning and building peer-learning habits [34]. However, there were instances of dissatisfaction when a team member did not actively participate and substantially contributed to question creation, which reflected adversely on team output. There are contradictory findings on peer collaboration from other studies; in one study, team cooperation toward item generation was perceived as unsatisfactory [27], whereas, in another study, willingness to collaborate with peers was agreed to by 86% of students [12]. During the ongoing COVID-19 pandemic, the rapid transition to distance learning provided the impetus to students from Queen’s University Belfast to create and share MCQs through Instagram to mutually enhance their learning [35]. Thus, it has been established that beneficial outcomes are a result of assessment-based peer-assisted learning.

Some students in this study perceived that the quality of the generated questions was inconsistent. They reported that some of their peers produced questions of low cognitive levels. This perceived weakness highlights the social regulation of learning, wherein the degree of achieved coregulation determines the enhancement of the ease and impact of learning on the individual and the group [2]. In contrast, in another study from a medical school in Cardiff, students were engaged in creating a question bank duly mentored and vetted by the content faculty. Within a 3-month period, 2800 tests had been attempted, indicating the popularity of the use of this learner resource [5]. The students who authored the MCQs in the Cardiff study were in their final year, which may have accounted for the higher quality of the generated questions.

The statistical reliability and validity of the survey tool provide a solid anchor for the results. A follow-up exercise based on the same framework in successive cohorts will further reinforce the tool’s reproducibility and the consistency and generalizability of the findings. Investigating the effectiveness of such an intervention can be performed by comparing before and after student performance scores. In one study, the follow-up scores on single best answer summative examinations correlated well, whereas performance on clinical examinations did not [10].

### Strengths, Limitations, and Future Directions

The key strength of this study lies in the integration of data derived from the mixed methods approach. This enables clarity on the aspects of building SRL that lend themselves to longitudinal replication, as well as identifies opportunities to dynamically respond to perceived challenges and weaknesses. The first limitation of this study was that the participating students were at an early stage of their medical school journey, which might have influenced their perceptions of the value of self-learning through assessment. It would be interesting to investigate in future studies whether the stage of learning plays a moderating effect on the students’ understanding and perception of the exercise and its impact by collecting perceptions from students at different stages of the program. Second, future research could use additional multiple-item formats to provide students with insights into their learning techniques. Finally, this study was limited in its application to a single course of 1 cohort of students. Hence, the generalizability of the findings is limited and can be remedied by making multicohort comparisons.

### Conclusions

Student-generated assessments in the form of AaL were perceived as viable, constructive, and stimulating educational exercises by the student authors. In the short term, the exercise constituted for the students a fun, challenging opportunity to dive deep into the content, be creative in designing questions, and improve examination-taking skills. Students expected long-term effects to include enhancement of critical thinking and the inculcation of student-regulated attributes of lifelong learning and peer collaboration, all of which are vital to the practice of medicine.

## Abbreviations

AaL: assessment as learning
AfL: assessment for learning
AoL: assessment of learning
LOA: learner-oriented assessment
MCQ: multiple-choice question
SRL: self-regulated learning

## Data Availability

The data collected in this study are included in the published manuscript and are available with the corresponding author.
